# Association of exposure to PM_2.5_-bound metals with premature rupture of membranes: a prospective cohort study

**DOI:** 10.3389/fpubh.2025.1603156

**Published:** 2025-06-16

**Authors:** Mengdan Liang, Liping Qiu, Biyun Lin, Zhehui Chen, Xiannuan Jiang, Mengying Xie, Xiaowei Xie, Hanbing Chen, Xiongkun He, Xiaoxiao Huang, Liang Lu, Lanlan Zhang, Hongjie Qiu, Yihong Chen, Junqi Wu, Xiaoxu Xie

**Affiliations:** ^1^Department of Epidemiology and Health Statistics, School of Public Health, Fujian Medical University, Fuzhou, China; ^2^The Collaboration Unit for State Key Laboratory of Infectious Disease Prevention and Control, Jiangxi Provincial Health Commission Key Laboratory of Pathogenic Diagnosis and Genomics of Emerging Infectious Diseases, Nanchang Center for Disease Control and Prevention, Nanchang, China; ^3^Department of Neonatal Intensive Care Unit, The First Affiliated Hospital, Fujian Medical University, Fuzhou, China; ^4^The Second Clinical Medical School, Nanchang University, Nanchang, China; ^5^Department of Urology, Harbin Medical University Cancer Hospital, Harbin, China; ^6^Hospital-Acquired Infection Control Department, Affiliated Jinhua Hospital, Zhejiang University School of Medicine, Jinhua, China; ^7^Department of Medical Imaging, Affiliated Jinhua Hospital, Zhejiang University School of Medicine, Jinhua, China; ^8^Department of Obstetrics and Gynecology, The First Affiliated Hospital, Fujian Medical University, Fuzhou, China; ^9^Department of Laboratory Medicine, Affiliated Jinhua Hospital, Zhejiang University School of Medicine, Jinhua, China; ^10^Clinical Research Unit, The Second Affiliated Hospital, Fujian Medical University, Quanzhou, China

**Keywords:** air pollution, particulate matter, metal, mixture, premature rupture of membranes

## Abstract

**Background:**

Exposure to PM_2.5_ has been linked to premature rupture of membranes (PROM). However, research on the effects of PM_2.5_-bound metals on the PROM is limited.

**Methods:**

Here, we investigated this relationship using data from 6090 pregnant women, estimating exposure to 11 PM_2.5_-bound metals throughout pregnancy. Cox models assessed associations between individual metals and PROM, while grouped weighted quantile sum regression (GWQS), quantile g-computation (Q-gcomp), and Bayesian kernel machine regression (BKMR) were used for metal mixtures.

**Results:**

Exposure to Al, Cd, Pb, Cr, Ni, Se, and Tl increased PROM risk, with hazard ratios ranging from 1.40 to 1.87. As and Mn were also correlated with PROM during specific trimesters. The GWQS model showed a 3% increased risk of PROM with metal mixture exposure (95% CI: 2%, 4%), mainly driven by Pb in the positive direction. The Q-gcomp model revealed a 5% increased risk (95% CI: 2%, 8%), also primarily due to Pb. In the BKMR model, Ni had the highest influence.

**Conclusion:**

Both individual metals and metal mixtures were associated with PROM, with Pb, Se, and Tl positively correlated with preterm PROM.

## Introduction

Premature rupture of membranes (PROM) is the rupture of gestational membranes prior to the onset of labor (1). Spontaneous PROM is estimated to affect approximately 8 to 10% of term pregnancies, which occur before the onset of uterine activity. Preterm PROM (pPROM), which is defined as PROM before 37 weeks of gestation, complicates 2 to 4% of all singleton pregnancies and a more substantial 7 to 20% of twin pregnancies and is a leading cause of perinatal mortality and morbidity, which is linked to 18 to 20% of perinatal deaths (2–5). PROM may lead to infection of the amniotic cavity in term newborns, and the greatest threat of PROM is preterm complications, the most common of which is neonatal respiratory distress (6).

PROM involves multiple pathways and factors, and its exact underlying mechanisms are currently unclear. PROM might be triggered by a cascade of biological mechanisms, notably inflammation, oxidative stress, cell apoptosis, and the activation of cytokines and matrix metalloproteinases (7, 8). Previous studies have shown that environmental factors are among the most significant triggers for PROM. A retrospective analysis of a cohort conducted in southern California revealed that high temperatures increase PROM risk (9), and PM_2.5_ sulfates, nitrates, ammonium, and organic compounds are linked to a greater risk of spontaneous premature rupture of membranes (sPROM) (10). A time series study conducted in Shanghai revealed that exposure to PM_2.5_, PM_10_, SO_2_, and CO is related to increased pPROM risk (11). Additionally, a cohort study conducted in Wuhan, China, revealed a notable correlation between exposure to PM_2.5_ during pregnancy and PROM (12). However, to the best of our knowledge, studies on the effects of PM_2.5_-bound metals on PROM are very limited.

Gestational age is a crucial factor that cannot be ignored in PROM, especially the effect of preterm birth on PROM. Previous studies have shown that exposure to air pollutants during pregnancy, such as PM_2.5_ and its components, PM_10_, NO_2_, CO, and O_3_, increases the risk of preterm birth, and that this effect varies depending on the exposure window (13–15). In this study, we paid special attention to the effect of gestational age on PROM. By analyzing the effects of PM_2.5_-bound metals on PROM in different exposure windows and stratifying term PROM and preterm PROM, we hope to gain a more in-depth understanding of the effects of air pollution on PROM, reveal the role of gestational age in this association of exposure to PM_2.5_-bound metals with PROM, and provide a scientific basis for the prevention and treatment of premature rupture of membranes.

Therefore, we used data from Jinhua Maternal and Child Health Care Hospital in Zhejiang Province to estimate (1) the relationships between maternal exposure to eleven PM_2.5_-bound metals, namely, aluminum (Al), arsenic (As), beryllium (Be), cadmium (Cd), plumbum (Pb), manganese (Mn), chromium (Cr), hydargyrum (Hg), nickel (Ni), selenium (Se), and thallium (Tl), and PROM in different exposure windows (i.e., throughout gestation, the first trimester, the second trimester, and the third trimester); (2) effects of PM_2.5_-bound metals on term PROM and preterm PROM, thereby revealing the potential effects of these metals on PROM at different gestational stages and their differences; and (3) the combined effects of eleven PM_2.5_-bound metal mixtures on PROM throughout pregnancy.

## Methods

### Study participants

The preliminary screening phase encompassed pregnant women who underwent antenatal examinations at Jinhua Maternal and Child Health Hospital, located in Zhejiang Province, China, between January 2017 and December 2019. A total of 6,090 pregnant women underwent antenatal checkups during the data collection period. Demographic information (e.g., maternal age, pre-pregnancy weight and height, educational attainment, family economy, nationality, and family address), lifestyle information (e.g., folic acid intake), and health status information (including medical and medication histories) were collected. Pregnant women were excluded if they resided outside Jinhua City, Zhejiang Province (156 cases), had missing data on their delivery date (869 cases), were carrying twin or multiple pregnancies (77 cases), had missing data on PROM (118 cases), had a gestational age of less than 20 or more than 47 weeks (3 cases), or experienced stillbirths (3 cases). Finally, 4,864 participants were included in the study (Supplementary Figure S1).

### Outcome: PROM

PROM is defined as spontaneous rupture of fetal membranes prior to delivery, irrespective of gestational age. In our study, PROM was defined after delivery. The gestation period in this study was from the last menstrual period to the date of labor.

### Exposure assessment

To assess exposure, PM_2.5_ samples were collected continuously for 7 days each month, from 10:00 AM to 9:00 AM the following day, using quartz fiber filters. Sampling was conducted using a medium-volume air sampler (model MSV6) manufactured by LECKEL in Germany, operating at a flow rate of 38.3 liters per minute. The metals bound to PM_2.5_, such as aluminum (Al), arsenic (As), beryllium (Be), cadmium (Cd), plumbum (Pb), manganese (Mn), chromium (Cr), hydargyrum (Hg), nickel (Ni), selenium (Se), and thallium (Tl), were analyzed using inductively coupled plasma-mass spectrometry (ICP-MS) equipment from Thermo Fisher Scientific (16). The mean value of PM_2.5_-bound metals for each month for each pregnant woman was calculated on the basis of the data collected on metals.

In our study, we categorized each participant’s exposure into four distinct periods: (1) the entire gestational period, encompassing the entire duration of pregnancy; (2) the first trimester, spanning from the beginning of pregnancy (week 1) to the end of the 12th week; (3) the second trimester, covering the period from the 13th to the 27th week of pregnancy; and (4) the third trimester, extending from the 28th week until the moment of delivery.

### Statistical analyses

Descriptive analysis of demographic characteristics and exposures of the entire population. The metal concentration is converted to a standard normal. The relevance between each metal is estimated via Spearman correlation.

We use the Cox regression to evaluate the risk ratio of PM_2.5_-bound metals to PROM using the number of gestation days as the time scale and use the Schoenfeld residual method to test the proportional hazards hypothesis. And we applied the False Discovery Rate (FDR) to consider the potential for type I error due to multiple comparisons. Covariates, including PM_2.5_ (continuous variable), maternal age (continuous variable), pre-pregnancy body mass index (BMI) (continuous variable), nationality (Han nationality, others, unknown), parity (nulliparous, multiparous, unknown), family economy (high, middle, low, unknown), educational attainment (junior high school or below, senior high school, college or above, unknown), sex (male, female, unknown) and season of conception (spring, summer, autumn, winter), were selected on the basis of existing studies (16–18). For missing values of covariates, we interpolated continuous covariates using the mean value and directly categorized the missing categorical variables into a new class. Moreover, the *E* value was used to evaluate the potential impact of unmeasured confounders on our study’s outcomes. The *E* value is defined as the smallest strength of the correlation in a hazard ratio (HR) scale, which depends on the measured covariate, whereas unmeasured confounding factors require an explanation of their association in terms of both treatment and outcome. The *E* value is concerned with the magnitude of the association of a confounder that may produce the same confounding bias as the observed association of the treatment outcome. We define the occurrence of PROM with a gestational age less than 37 weeks as preterm PROM, and that with a gestational age of 37 weeks or more as term PROM. Stratified analyses of term PROM and preterm PROM were performed to explore the effects of PM_2.5_-bound metals on PROM at different gestational ages. To explore the exposure-response relationship between PM_2.5_-bound metals and PROM, we plotted restricted cubic spline curves with 3 knots at the 10th, 50th and 90th percentiles.

We used the group weight quantile sum model (GWQS), quantile g-computation (Q-gcomp), and Bayesian kernel machine regression (BKMR) to assess the joint effect of PM_2.5_-bound metals on the PROM. GWQS may consider multiple groups of exposures to estimate as many associations with outcomes as possible for different magnitudes and directions (19). In our study, we assessed the overall effect of PM_2.5_-bound metals on PROM by dividing them into positive and negative groups on the basis of the results of Cox regression. Like WQS regression, Q-gcomp is characterized by simplicity of interpretation and ease of calculation, but it does not assume directional homogeneity (20). BKMR makes use of kernel regression, leveraging the Bayesian algorithm, to address high-dimensional parameter spaces as well as nonlinear and nonadditive data based on the Bayesian algorithm, thus providing flexibility in modeling the combined effects of the mixtures (21). BKMR was implemented through the “bkmr” package in R. Comparison of the three models can be found in Supplementary Table S1.

In the sensitivity analyses, (a) we tested for correlations via the accelerated failure time model. (b) We used Cox proportional risk models stratified by several basic characteristics to identify susceptible populations, including maternal age (≤24, 25–29, ≥30), infant’s sex (male, female), pre-pregnancy body mass index (BMI) (<18.5, 18.5–23.9, ≥24), parity (nulliparous, multiparous), educational attainment (junior high school or below, senior high school, college or above). (c) We stratify by some basic characteristics on the basis of grouped WQS to examine the robustness of the GWQS results. All the analyses were conducted with R version 4.1.3.

## Results

### Descriptive statistics

Table 1 summarizes the characteristics of the study population. A total of 4,864 participants with 194 (4.0%) PROM cases were included. The mean age and pre-pregnancy BMI of the mothers were 29.39 and 21.41, respectively. Most of the study subjects had a median family economy (95.5%). More than 50% of mothers had a university education or above (58.8%). The vast majority of mothers were Han Chinese (96.3%).

**Table 1:** Descriptive statistics of the study population.

Characteristics	Total pregnancies (*n* = 4,864)	Non-PROMs (*n* = 4,670)	PROMs (*n* = 194)
Maternal age, year^a^	29.39 (4.80)	29.42 (4.81)	28.74 (4.47)
Pre-pregnancy BMI, kg/m^2 a^	21.41 (2.85)	21.39 (2.85)	21.80 (2.77)
Infant’s sex, *n* (%)			
Male	2,302 (47.3%)	2,204 (47.2%)	98 (50.5%)
Female	2,138 (44.0%)	2058 (44.1%)	80 (41.2%)
Unknown	424 (8.7%)	408 (8.7%)	16 (8.2%)
Family economy, *n* (%)			
High	202 (4.2%)	190 (4.1%)	12 (6.2%)
Middle	4,647 (95.5%)	4,466 (95.6%)	181 (93.3%)
Low	2 (0.0%)	2 (0.0%)	0 (0.0%)
Unknown	13 (0.3%)	12 (0.3%)	1 (0.5%)
Educational level, *n* (%)			
Junior high school or below	603 (12.4%)	590 (12.6%)	13 (6.7%)
Senior high school	1,393 (28.6%)	1,340 (28.7%)	53 (27.3%)
College or above	2,858 (58.8%)	2,730 (57.5%)	128 (66.0%)
Unknown	10 (0.2%)	10 (0.2%)	0 (0.0%)
Ethnic, *n* (%)			
Han nationality	4,686 (96.3%)	4,499 (96.3%)	187 (96.4%)
Others	177 (3.6%)	170 (3.6%)	7 (3.6%)
Unknown	1 (0.0%)	1 (0.0%)	0 (0.0%)
Parity, *n* (%)			
Nulliparous	2,296 (47.2%)	2,184 (46.8%)	112 (57.7%)
Multiparous	2,567 (52.8%)	2,485 (53.2%)	82 (42.3%)
Unknown	1 (0.0%)	1 (0.0%)	0 (0.0%)
Season of conception, *n* (%)			
Spring	1,195 (24.6%)	1,148 (24.6%)	47 (24.2%)
Summer	1,071 (22.0%)	1,037 (22.2%)	34 (17.5%)
Autumn	1,269 (26.1%)	1,229 (26.3%)	40 (30.6%)
Winter	1,329 (27.3%)	1,256 (26.9%)	73 (37.6%)

BMI, body mass index. ^a^Data are expressed as mean (standard deviation).

Table 2 describes the distribution of PM_2.5_-bound metal exposure levels among the study population during pregnancy. The average concentrations of Al, As, Be, Cd, Pb, Mn, Cr, Hg, Ni, Se, and Tl throughout pregnancy were 27.74 ng/m^3^, 3.16 ng/m^3^, 0.21 ng/m^3^, 15.43 ng/m^3^, 29.63 ng/m^3^, 15.67 ng/m^3^, 3.24 ng/m^3^, 2.75 ng/m^3^, 3.80 ng/m^3^, 6.66 ng/m^3^ and 0.71 ng/m^3^, respectively. Supplementary Figure S2 shows Spearman correlation coefficients between PM_2.5_-bound metal exposure throughout pregnancy. Al and Be, Cd, Pb, Ni, Se, As and Pb, Mn, Cr, Be and Cd, Pb, Cd and Pb, Ni, Se, Pb and Mn, Mn and Cr, Ni and Se were strongly correlated.

**Table 2:** Summary statistics (means ± SDs) of the mean PM_2.5_-bound metal exposure of the study subjects throughout pregnancy.

Metals	Total pregnancies (*n* = 4,864)	Non-PROMs (*n* = 4,670)	PROMs (*n* = 194)
Al, ng/m^3^	27.74 ± 6.77	27.71 ± 6.75	28.31 ± 7.29
As, ng/m^3^	3.16 ± 0.46	3.16 ± 0.46	3.09 ± 0.40
Be, ng/m^3^	0.21 ± 0.10	0.21 ± 0.10	0.19 ± 0.10
Cd, ng/m^3^	15.43 ± 3.33	15.42 ± 3.32	15.60 ± 3.51
Pb, ng/m^3^	29.63 ± 5.78	29.63 ± 5.78	29.80 ± 5.96
Mn, ng/m^3^	15.67 ± 4.10	15.71 ± 4.12	14.79 ± 3.36
Cr, ng/m^3^	3.24 ± 0.67	3.23 ± 0.67	3.39 ± 0.58
Hg, ng/m^3^	2.75 ± 0.71	2.75 ± 0.71	2.86 ± 0.65
Ni, ng/m^3^	3.80 ± 0.77	3.79 ± 0.77	3.99 ± 0.85
Se, ng/m^3^	6.66 ± 3.06	6.63 ± 3.06	7.26 ± 3.09
Tl, ng/m^3^	0.71 ± 0.15	0.71 ± 0.15	0.75 ± 0.12

Al, aluminum; As, arsenic; Be, beryllium; Cd, cadmium; Pb, plumbum; Mn, manganese; Cr, chromium; Hg, hydrargyrum; Ni, nickel; Se, selenium; Tl, thallium.

### Single metal and PROM

The associations between individual PM_2.5_-bound metals and preterm rupture of membranes (PROM), along with their corresponding *E* values for each trimester and the entire pregnancy, are presented in Table 3. The results of proportional hazards hypothesis test by the Schoenfeld residual method showed that all models met the equal-proportionality assumption (*p* > 0.05) (Supplementary Figure S3). We can observe that eleven PM_2.5_-bound metals were associated with the PROM, but this association varies according to the exposure window. During the entire pregnancy, Al, Cd, Pb, Cr, Ni, Se, and Tl increased PROM risk (HRs ranging from 1.40 to 1.87). Be decreased risk (HR = 0.79). In the first trimester, Al, As, and Pb increased risk (HRs from 1.49 to 2.06). In the second trimester, Cd, Ni, and Se elevated risk (HRs from 1.36 to 1.44). In the third trimester, Pb, Cr, and Tl increased risk (HRs from 2.04 to 4.12), while Mn and Hg decreased risk (HRs of 0.47 and 0.63). *E* values were in the range of 1.00 ~ 7.71, which indicated that an unmeasured confounder beyond those included in our models would need to have a hazard ratio of ≥7.71 relative to the outcome to completely diminish the observed effects in our study.

**Table 3:** Premature rupture hazard ratios (estimates and 95% confidence intervals) and their *E* values associated with individual PM_2.5_-bound metals in different gestational trimesters based on the Cox proportional hazards model.

Metals	Entire pregnancy			1st trimester			2nd trimester			3rd trimester		
	HR (95% CI)	*E*-value	*P_FDR_*-value	HR (95% CI)	*E*-value	*P_FDR_*-value	HR (95% CI)	*E*-value	*P_FDR_*-value	HR (95% CI)	*E*-value	*P_FDR_*-value
Al	1.45 (1.14, 1.85)	2.26	0.005	1.49 (1.13, 1.97)	2.34	0.030	1.20 (0.89, 1.62)	1.69	0.367	1.00 (0.73, 1.37)	1.00	0.990
As	1.51 (0.99, 2.30)	2.39	0.061	2.06 (1.21, 3.52)	3.54	0.030	1.09 (0.46, 2.58)	1.40	0.929	0.99 (0.49, 2.01)	1.11	0.990
Be	0.79 (0.65, 0.96)	1.85	0.028	0.77 (0.62, 0.97)	1.92	0.067	0.99 (0.80, 1.24)	1.11	0.949	1.00 (0.82, 1.22)	1.00	0.990
Cd	1.40 (1.08, 1.81)	2.15	0.017	0.97 (0.78, 1.22)	1.21	0.933	1.44 (1.10, 1.87)	2.24	0.027	0.70 (0.49, 1.02)	2.21	0.121
Pb	1.50 (1.17, 1.93)	2.37	0.003	1.83 (1.19, 2.81)	3.06	0.030	1.26 (0.81, 1.95)	1.83	0.416	4.12 (2.13, 7.96)	7.71	< 0.001
Mn	0.74 (0.44, 1.23)	2.04	0.287	1.23 (0.85, 1.80)	1.76	0.503	1.63 (0.96, 2.74)	2.64	0.150	0.47 (0.31, 0.72)	3.68	0.001
Cr	1.47 (1.03, 2.10)	2.30	0.048	0.97 (0.74, 1.28)	1.21	0.933	0.89 (0.67, 1.17)	1.50	0.472	2.14 (1.57, 2.91)	3.70	< 0.001
Hg	0.93 (0.72, 1.20)	1.36	0.543	0.96 (0.77, 1.20)	1.25	0.933	1.23 (0.99, 1.52)	1.76	0.150	0.63 (0.48, 0.83)	2.55	0.002
Ni	1.40 (1.16, 1.69)	2.15	0.002	1.01 (0.83, 1.22)	1.11	0.933	1.36 (1.10, 1.69)	2.06	0.027	0.95 (0.74, 1.22)	1.29	0.990
Se	1.64 (1.23, 2.19)	2.66	0.002	1.04 (0.85, 1.27)	1.24	0.933	1.40 (1.11, 1.76)	2.15	0.027	1.03 (0.83, 1.26)	1.21	0.990
Tl	1.87 (1.42, 2.46)	3.15	<0.001	1.17 (1.00, 1.38)	1.62	0.112	0.87 (0.71, 1.07)	1.56	0.342	2.04 (1.57, 2.65)	3.50	< 0.001

HR, hazard ratio; CI, confidence interval; Al, aluminum; As, arsenic; Be, beryllium; Cd, cadmium; Pb, plumum; Mn, manganese; Cr, chromium; Hg, hydargyrum; Ni, nickel; Se, selenium; Tl, thallium. Hazard ratio (HR) of PROM risk per standard deviation increment in metal concentrations. All models were adjusted for PM_2.5_, maternal age, maternal educational level, family economy, pre-pregnancy BMI, sex, ethnicity, parity, and season of conception. *p* values were corrected by FDR method.

The results in Table 4 show most PM_2.5_-bound metals were more strongly associated with preterm PROM than term PROM. Pb, Se and Tl were significantly linked to preterm PROM, with HRs of 2.77 (95% CI: 1.02, 7.54), 2.94 (95% CI: 1.07, 8.06) and 2.35 (95% CI: 1.11, 4.99), respectively.

**Table 4:** Hazard ratios (estimates and 95% confidence intervals) and their *P* values for term PROM and preterm PROM associated with individual PM_2.5_-bound metals based on the Cox proportional hazards model.

Metals	Preterm PROM (*n* = 225)		Term PROM (*n* = 4,639)	
	HR (95% CI)	*P*-value	HR (95% CI)	*P*-value
Al	1.99 (0.86, 4.61)	0.108	1.29 (0.98, 1.69)	0.067
As	2.68 (0.41, 17.38)	0.300	1.33 (0.84, 2.13)	0.228
Be	0.95 (0.51, 1.76)	0.868	0.83 (0.67, 1.04)	0.106
Cd	1.87 (0.89, 3.94)	0.098	1.22 (0.92, 1.63)	0.171
Pb	2.77 (1.02, 7.54)	0.046	1.33 (1.00, 1.76)	0.047
Mn	0.16 (0.02, 1.39)	0.096	0.77 (0.44, 1.33)	0.343
Cr	1.00 (0.41, 2.46)	0.996	1.34 (0.93, 1.93)	0.115
Hg	0.79 (0.46, 1.36)	0.397	1.10 (0.82, 1.47)	0.542
Ni	1.86 (0.97, 3.56)	0.063	1.28 (1.04, 1.56)	0.017
Se	2.94 (1.07, 8.06)	0.036	1.42 (1.04, 1.95)	0.030
Tl	2.35 (1.11, 4.99)	0.026	1.57 (1.18, 2.10)	0.002

HR, hazard ratio; CI, confidence interval; Al, aluminum; As, arsenic; Be, beryllium; Cd, cadmium; Pb, plumum; Mn, manganese; Cr, chromium; Hg, hydargyrum; Ni, nickel; Se, selenium; Tl, thallium. Hazard ratio (HR) of PROM risk per standard deviation increment in metal concentrations. All models were adjusted for PM_2.5_, maternal age, maternal educational level, family economy, pre-pregnancy BMI, sex, ethnicity, parity, and season of conception.

**Figure 1:**
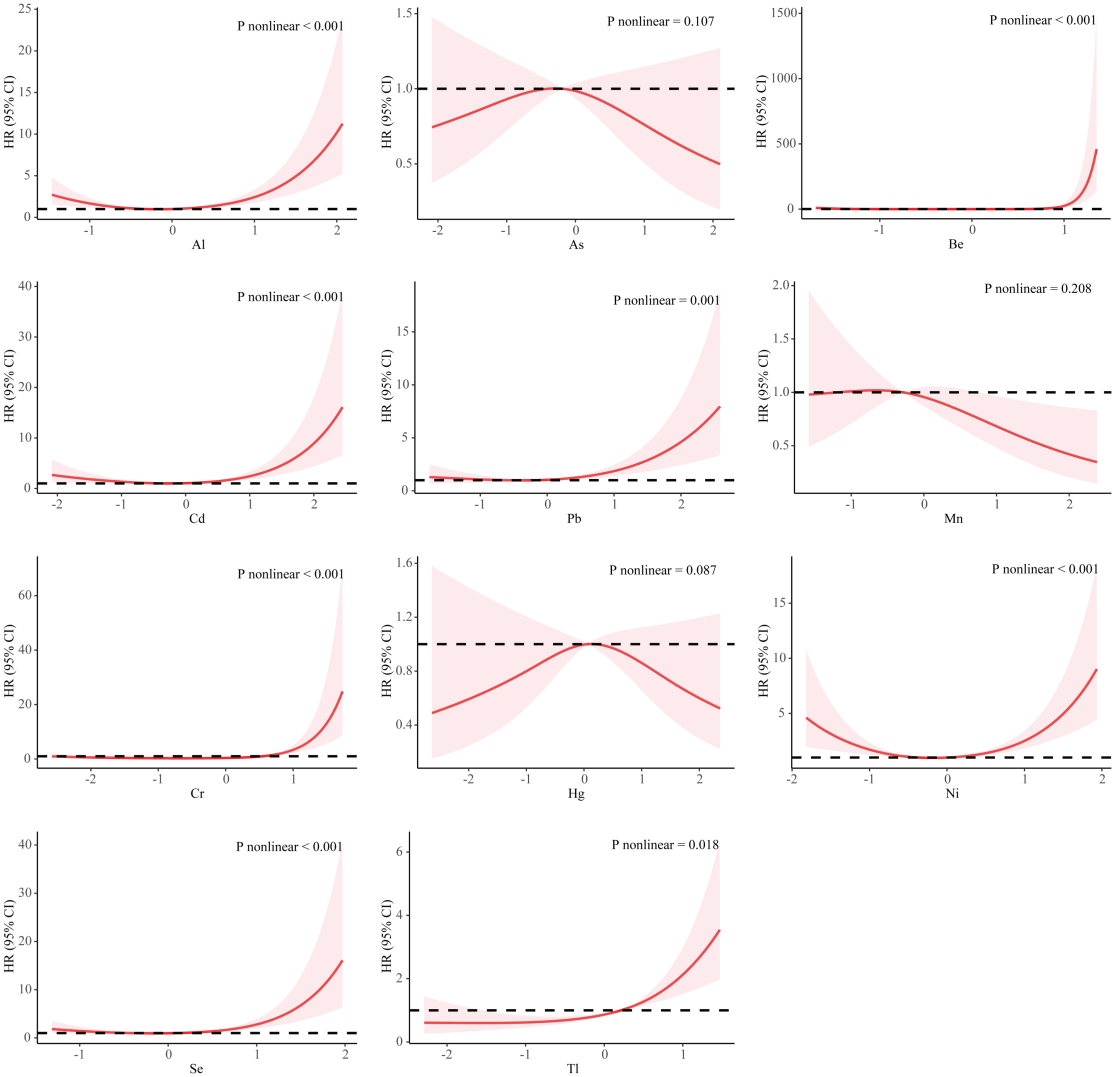
Association between metals and the premature rupture of membranes based on the restricted cubic spline model. The covariates that were adjusted included PM_2.5_, maternal age, infant sex, pre-pregnancy BMI, educational level, family economy, ethnicity, parity, and season of conception. The metal concentration is converted to a standard normal. HR, hazard ratio; CI, confidence interval; Al, aluminum; As, arsenic; Be, beryllium; Cd, cadmium; Pb, plumbum; Mn, manganese; Cr, chromium; Hg, hydride; Ni, nickel; Se, selenium; Tl, thallium.

The exposure-response relationship between individual PM_2.5_-bound metals and PROM was fitted by a restricted cubic spline function with a knot of 3 knots. As, Mn and Hg showed a linear relationship with PROM (*p* > 0.05), with a monotonically increasing trend for Tl. Al, Cd, Pb, Ni, and Se showed an approximately U-shaped relationship with PROM, with the risk of PROM decreasing and then increasing as the concentration of pollutants increased. As Mn and Hg exhibited an inverted U-shaped relationship, the risk of PROM increased and then decreased with increasing pollutant concentration, whereas Be and Cr tended to increase within a certain range (Figure 1).

### Metal mixture and PROM

**Figure 2:**
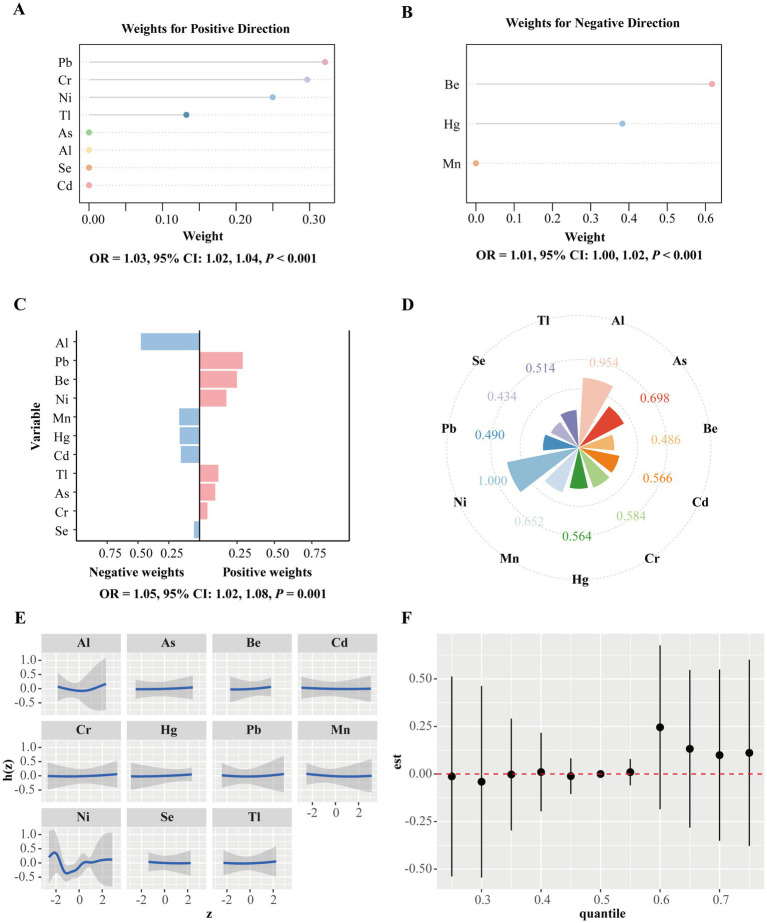
Mixtures of metals and the premature rupture of membranes. **(A)** The GWQS model constrained the effect of exposure to PM_2.5_-bound metal mixtures and the outcome in a positive direction. **(B)** The GWQS model constrained the effect of metal mixture exposure and the outcome in a negative direction. **(C)** Weight distributions for the negative and positive directions based on Q-gcomp. This model adjusted for PM_2.5_, maternal age, infant sex, pre-pregnancy BMI, educational level, family economy, ethnicity, parity, and season of conception. **(D)** The BKMR model calculates the conditional posterior inclusion probabilities (PIPs) to determine the relative importance of individual metal exposure. **(E)** Exposure response curves for eleven metals when the concentrations of the other metals are at a median level. **(F)** Metal mixture associations from the BKMR model were combined and calculated by comparing the kernel function values when all metals were at specific percentiles (x-axis) with those when all metals were fixed at the 50th percentile. The BKMR model adjusts for maternal age, sex, pre-pregnancy BMI, educational level, family economy, ethnicity, parity, and season of conception. GWQS, group weight quantile sum; Q-gcomp, quantile g-computation; BKMR, Bayesian kernel machine regression; Al, aluminum; As, arsenic; Be, beryllium; Cd, cadmium; Pb, plumbum; Mn, manganese; Cr, chromium; Hg, hydrargyrum; Ni, nickel; Se, selenium; Tl, thallium.

The mixed analysis results based on the GWQS model, Q-gcomp and BKMR are shown in Figure 2. Throughout the gestational period, positive and negative groups were generated according to the results of Cox regression. In the GWQS model, a mixture of metals in the positive direction increased the risk of PROM by 3% (95% CI: 2, 4%), and the positive weights associated with PROM were driven primarily by Pb, followed by Cr, Ni, Tl, As, Al, Se, and Cd (Figure 2A). In the negative direction, the PROM risk increased by −1% (95% CI: −2, 0%), and Be is the most heavily weighted in the negative direction, followed by Hg and Mn (Figure 2B). The results of Q-gcomp show that for every quarter increase in all the metal mixtures, the incidence of PROM increased by 5% (95% CI: 2, 8%), where the positive direction was driven mainly by Pb and the negative direction was driven mainly by Al (Figure 2C). The result of BKMR reveals that Ni had the largest PIP (1.000), followed by Al (0.954) (Figure 2D). Figure 2E illustrates the nonlinear relationships for each mixture metal. The joint effect of PM_2.5_-bound metals on PROM was greater at the 60th percentile and above, and this effect was statistically significant (Figure 2F).

### Sensitivity analyses

Supplementary Table S1 and Supplementary Figures S4, S5 show the results of the sensitivity analyses. Supplementary Table S1 shows the association between PROM and individual PM_2.5_-bound metals throughout gestation on the basis of accelerated failure time models. The results reveal that maternal exposure to Al, Cd, Pb, Cr, Ni, Se, and Tl throughout gestation; to Al, As, Pb and Tl in early pregnancy; to Cd, Ni, and Se in mid-pregnancy; and to Pb, Cr and Tl in late pregnancy increased the incidence of PROM.

Supplementary Figure S4 shows the results of the stratified analyses based on Cox proportional risk regression. Exposure to Se, Ni, and Tl during pregnancy and mothers aged <30 years were associated with increased PROM risk. In addition, exposure to Cr and Mn increased the susceptibility to develop PROM in pregnant women aged ≤24 years and ≥30 years. For Al, Be, Pb and Cd, the susceptibility period was 25–29 years. There were differences in susceptibility to different metal exposures according to the infant’s sex. Compared with males, females were more susceptible to most metals. Pregnant women with pre-pregnancy BMIs between 18.5 and 23 kg/m^2^ were susceptible to the occurrence of PROM. Exposure to Al, Cr, Se, Tl, and Ni during pregnancy was associated with a higher PROM risk in primiparous women. The risk of PROM was greater when the mother’s educational level was college and above. Supplementary Figure S5 shows the results of the stratified analyses based on GWQS. The results show that the GWQS results were almost robust.

## Discussion

In this study, we investigated the nexus between exposure to PM_2.5_-bound metals and PROM throughout gestation and during the entire trimester. Overall, we found that exposure to eleven PM_2.5_-bound metals during gestation is associated with PROM, but the association varies across exposure windows. Exposure to Al, As, Cd, Pb, Cr, Ni, Se, and Tl increased the risk of PROM. The critical window for Al, As and Pb was early pregnancy; for Cd, Ni, and Se, it was mid-pregnancy; and for Pb, Cr and Tl, it was late pregnancy. Whereas exposure to Mn decreases the risk of PROM, the critical window is late pregnancy for Mn. In addition, our study revealed that exposure to Pb, Se, and Tl during pregnancy increased the risk of preterm PROM. The GWQS model and quantile g-computation model were statistically significant. The GWQS model is driven mainly in the positive direction, with Pb having the maximum weight in the positive direction and Be having the maximum weight in the negative direction. Similar results were reported in the quantile g-computational model, which is driven mainly in the positive direction, with Pb having the greatest weight in the positive direction and Al having the greatest weight in the negative direction. In the BKMR model, the joint effect of PM_2.5_-bound metals on PROM was greater at the 60th percentile and above, and this effect was statistically significant, and Ni accounted for the largest weight.

With rapid economic development and industrialization, heavy metal pollution has become an important issue in China. PM_2.5_ is a carrier of many heavy metals and has a greater ability to carry them, posing a greater risk to public wellness (22). Earlier studies have indicated that contact with heavy metals is correlated with various clinical obstetric complications and negative pregnancy outcomes, including gestational diabetes (23), preeclampsia (24), low birth weight (25, 26) and preterm delivery (27). However, the association between heavy metals and PROM has not been widely substantiated by research. In this study, exposure to Al, As, Cd, Pb, Cr, Ni, Se, and Tl during pregnancy was found to be positively associated with PROM. A comprehensive birth cohort from China investigated the link between maternal urinary chromium levels and PROM, revealing a positive correlation between the two after accounting for other factors (28). Another birth cohort study in China revealed a positive association between elevated maternal Pb levels and PROM (17). Turning to the potential mechanisms, several studies have shown that exposure of pregnant women to environmental factors (e.g., air pollution) can increase PROM through oxidative stress (OS) and inflammatory response mechanisms (18, 29). Studies involving both humans and animals have indicated that heavy metal exposure during pregnancy elevates oxidative stress (30–34). Specifically, reactive oxygen species (ROS), which are continuously generated within the body, have been implicated in collagen damage, potentially leading to tearing in the chorionic amniotic sac (35). As a result, OS due to increased ROS may weaken collagen intensity and elasticity and lead to premature membrane damage by altering intracellular biology and membrane lipid peroxidation, which reduces membrane fluidity and impairs membrane barrier function, leading to PROM (35–37). Furthermore, the intravascular inflammatory response during pregnancy is reportedly linked to rupture of fetal membranes, which may give rise to PROM (38, 39). While some heavy metals can elicit inflammatory responses through increasing the expression of proinflammatory cytokines (40, 41), thereby increasing the risk of PROM.

Manganese (Mn), a vital trace element in human biology, is crucial for the growth and development of the fetus (42–44). A nationwide nutrition and health survey conducted across the United States revealed that pregnant women had higher blood concentrations of Mn than nonpregnant women did, suggesting that Mn plays a vital role during gestation (45). A case–control study involving a birth cohort in Beijing suggested that elevated plasma Mn concentrations in late pregnancy are relevant to a high hazard of spontaneous preterm birth (46). Our study revealed that exposure to Mn throughout gestation and in late pregnancy reduced the risk of PROM. Because there are no studies on the relationship between Mn and PROM, more research is needed to substantiate our findings.

We used three models for mixture analysis. Each of the components of a mixture may act independently, synergistically, or antagonistically, or they may have no effect on the health outcome of interest. Focusing on the mixture as a whole is beneficial because it provides simple reasoning, integrates multiple exposures that may come from similar sources, and often directly maps to the effects of potential public health interventions (20). In our study, the results of all three mixture models found that joint exposure to PM_2.5_-bound metals increased the risk of PROM. However, Pb had the greatest weight in the positive direction in GWQS model and Q-gcomp model, whereas in the BKMR model, the greatest contribution was made by Tl, which may be due to differences in the rationale and assumptions of these models. The GWQS model and the Q-gcomp model identify independent effects by distinguishing between positive and negative directions; whereas the BKMR model focuses on capturing complex interactions without distinguishing between positive and negative directions (47).

There are several strengths in this study. First, the consecutive monitoring of multiple PM_2.5_-bound metals over an extended duration enhances our understanding of their effects on PROM. Second, the inclusion of a large sample size provides sufficient statistical power to detect the influence of PM_2.5_-bound metal exposure on PROM, allowing for the adjustment of potential confounders and estimating the role of unmeasured confounders by calculating *E* values. Third, we used three mixture models to assess the collective impact of eleven PM_2.5_-bound metals on the PROM, which has not been done before.

However, this study also has limitations. Notably, the evaluation of PM_2.5_-bound metal exposure is grounded in measurements at fixed locations, which do not consider individual spatial differences and may lead to exposure misclassification. Moreover, the lack of data limits our ability to consider indoor air pollution and mothers’ patterns of activity over time in our analysis. Second, we could not assess the concentrations of metals in pregnant women’s blood or excreted urine, which would indicate the internal exposure dose. Therefore, the level of metals bound to PM_2.5_ may not accurately represent the actual exposure experienced by pregnant women. Third, owing to limited information on the data collected, some lifestyles that have an impact on outcomes, such as smoking and alcohol consumption, were not considered in the analyses. Finally, because we had access only to monthly air pollution data, we used only crude definitions for the entire gestation period and trimesters without investigating more specific exposure windows or any acute effects of PM_2.5_-bound metal exposure, including associations on a weekly or daily basis. On the basis of monthly exposure data, our results for specific pregnancies may not be accurate enough.

In conclusion, exposure to PM_2.5_-bound metals during pregnancy, both individually and as a mixture, was associated with PROM. Specific metals such as Al, As, Cd, Pb, Cr, Ni, Se, and Tl showed positive correlations, while Mn had a negative association. Notably, Pb, Se, and Tl were linked to a higher risk of preterm PROM. Further research is required to confirm these associations and explore strategies to minimize exposure during pregnancy to protect the health of mothers and fetuses. Additionally, in the past, the search for predictive markers of neonatal neurological outcome in preterm PROM has been limited to studies examining the relationship between various inflammatory mediators and prognosis, whereas in light of the results of the present study, it may be possible in the future to further test pregnant women for PM_2.5_-bound metals, which could provide new clues in the search for more accurate neonatal neurological prognostic markers.

## Data Availability

The original contributions presented in the study are included in the article/Supplementary material, further inquiries can be directed to the corresponding authors.

## Author’s note

No research has investigated the effects of particulate matter-bound metals on the premature rupture of membranes. We report that ambient particulate matter-bound metals potentially affect the premature rupture of membranes.
