# Supplementary material for: Association of exposure to PM2.5-bound metals with premature rupture of membranes: a prospective cohort study
Source: Front Public Health. 2025 Jun 16;13:1603156. doi: 10.3389/fpubh.2025.1603156 (PMC12207744; doi:10.3389/fpubh.2025.1603156)
Supplement: Supplementary file 1 [file Table_1.docx]

**Supplementary Online Content**

**Association of exposure to PM_2.5_-bound metals with premature rupture of membranes: a prospective cohort study**

**Supplemental Table S1** The comparison of the advantages and disadvantages for the three mixture models.

**Supplemental Table S2** Premature rupture of membranes hazard ratios (estimate and 95% confidence interval) and their *P*-values associated with individual PM_2.5_-bound metals in different gestational trimesters based on Accelerated Failure Time Model.

**Supplemental Figure S1** Flowchart of the participants selection.

**Supplemental Figure S2** Spearman correlations between metals.

**Supplemental Figure S3** Residual plot for proportional hazards hypothesis test using Schoenfeld residual method.

**Supplemental Figure S4** Forest maps for Cox stratified analyses.

**Supplemental Figure S5** Forest maps for GWQS stratified analyses.

Table S1

The comparison of the advantages and disadvantages for the three mixture models^[1-4]^.

| Models | GWQS | Q-gcomp | BKMR |
| --- | --- | --- | --- |
| Advantages | (1) Regression analyses that integrate multiple exposure factors into a composite index can be effective in assessing the overall effect of complex mixed exposures on health; (2) The ability to group multiple relevant exposure factors and assess the combined effect of each group. | (1) Allows for nonlinear effects between exposure and outcome variables; (2) Allows simultaneous estimation of effects in different directions. | (1) Detecting interactions among chemicals;  (2) Estimating nonlinear exposure-response relationships. |
| Limitations | (1) Converting continuous exposure data to quartile form may result in partial loss of data information; (2) It is usually assumed that all exposure factors within the same group affect the outcome in the same direction, which may affect the accuracy of the results if there is actually an effect in the opposite direction. | (1) Converting continuous exposure data to quartile form may result in partial loss of data information; (2) When the base model is not smooth, the edge structure model may not adequately capture the exposure response function. | (1) Restricted in evaluating the impacts of exposure to chemical mixtures that include both elevated and low concentrations; (2) Higher computational effort, especially if the sample size is large, may require longer time and more resources. |

** Abbreviations: GWQS, Group weight quantile sum model; Q-gcomp, Quantile g-computation model; BKMR, Bayesian kernel machine regression model.

Table S2

Premature rupture of membranes hazard ratios (estimate and 95% confidence interval) and their *P*-values associated with individual PM_2.5_-bound metals in different gestational trimesters based on Accelerated Failure Time Model.

| Metals | Entire pregnancy | |  | 1st trimester | |  | 2nd trimester | |  | 3rd trimester | |
| --- | --- | --- | --- | --- | --- | --- | --- | --- | --- | --- | --- |
|  | HR (95% CI) | *P*-value |  | HR (95% CI) | *P*-value |  | HR (95% CI) | *P*-value |  | HR (95% CI) | *P*-value |
| Al | 0.01 (0.00, 0.21) | 0.003 |  | 0.01 (0.00, 0.24) | 0.005 |  | 0.11 (0.00, 4.53) | 0.248 |  | 1.05 (0.02, 45.81) | 0.982 |
| As | 0.01 (0.00, 1.02) | 0.051 |  | 0.00 (0.00, 0.12) | 0.010 |  | 0.30 (0.00, 10473.21) | 0.822 |  | 0.95 (0.00, 4475.81) | 0.990 |
| Be | 18.14 (1.61, 204.82) | 0.019 |  | 21.86 (1.47, 324.67) | 0.025 |  | 1.18 (0.08, 16.62) | 0.904 |  | 1.01 (0.09, 11.23) | 0.993 |
| Cd | 0.01 (0.00, 0.36) | 0.009 |  | 1.34 (0.09, 21.02) | 0.836 |  | 0.01 (0.00, 0.27) | 0.006 |  | 79.84 (0.90, 7064.86) | 0.055 |
| Pb | 0.01 (0.00, 0.15) | 0.002 |  | 0.00 (0.00, 0.14) | 0.007 |  | 0.08 (0.00, 16.80) | 0.356 |  | 0.00 (0.00, 0.00) | < 0.001 |
| Mn | 49.46 (0.10, 25542.98) | 0.221 |  | 0.09 (0.00, 8.22) | 0.292 |  | 0.00 (0.00, 1.09) | 0.053 |  | 7570.88 (49.11, 1167183.00) | 0.001 |
| Cr | 0.01 (0.00, 0.61) | 0.029 |  | 1.21 (0.05, 31.93) | 0.907 |  | 4.91 (0.18, 136.26) | 0.348 |  | 0.00 (0.00, 0.00) | < 0.001 |
| Hg | 2.41 (0.11, 51.12) | 0.573 |  | 1.70 (0.11, 26.37) | 0.704 |  | 0.09 (0.01, 1.18) | 0.067 |  | 205.37 (8.28, 5091.84) | 0.001 |
| Ni | 0.02 (0.00, 0.15) | < 0.001 |  | 0.86 (0.09, 8.68) | 0.899 |  | 0.02 (0.00, 0.28) | 0.004 |  | 2.12 (0.10, 43.01) | 0.626 |
| Se | 0.00 (0.00, 0.08) | 0.001 |  | 0.61 (0.05, 7.11) | 0.691 |  | 0.02 (0.00, 0.27) | 0.004 |  | 0.84 (0.07, 10.26) | 0.890 |
| Tl | 0.00 (0.00, 0.01) | < 0.001 |  | 0.14 (0.02, 0.98) | 0.047 |  | 6.11 (0.51, 72.69) | 0.152 |  | 0.00 (0.00, 0.00) | < 0.001 |

** Abbreviations: HR, hazard ratios; CI, confidence interval; Al, Aluminium; As, Arsenic; Be, Beryllium; Cd, Cadmium; Pb, Plumbum; Mn, Manganese; Cr, Chromium; Hg, Hydrargyrum; Ni, Nickel; Se Selenium; Tl, Thallium. Hazard ratio (HR) of PROM risk per standard deviation increment in metal concentrations. All models were adjusted for PM_2.5_, maternal age, maternal educational level, family economy, pre-pregnancy BMI, infant’s sex, ethnic, parity, season of conception.


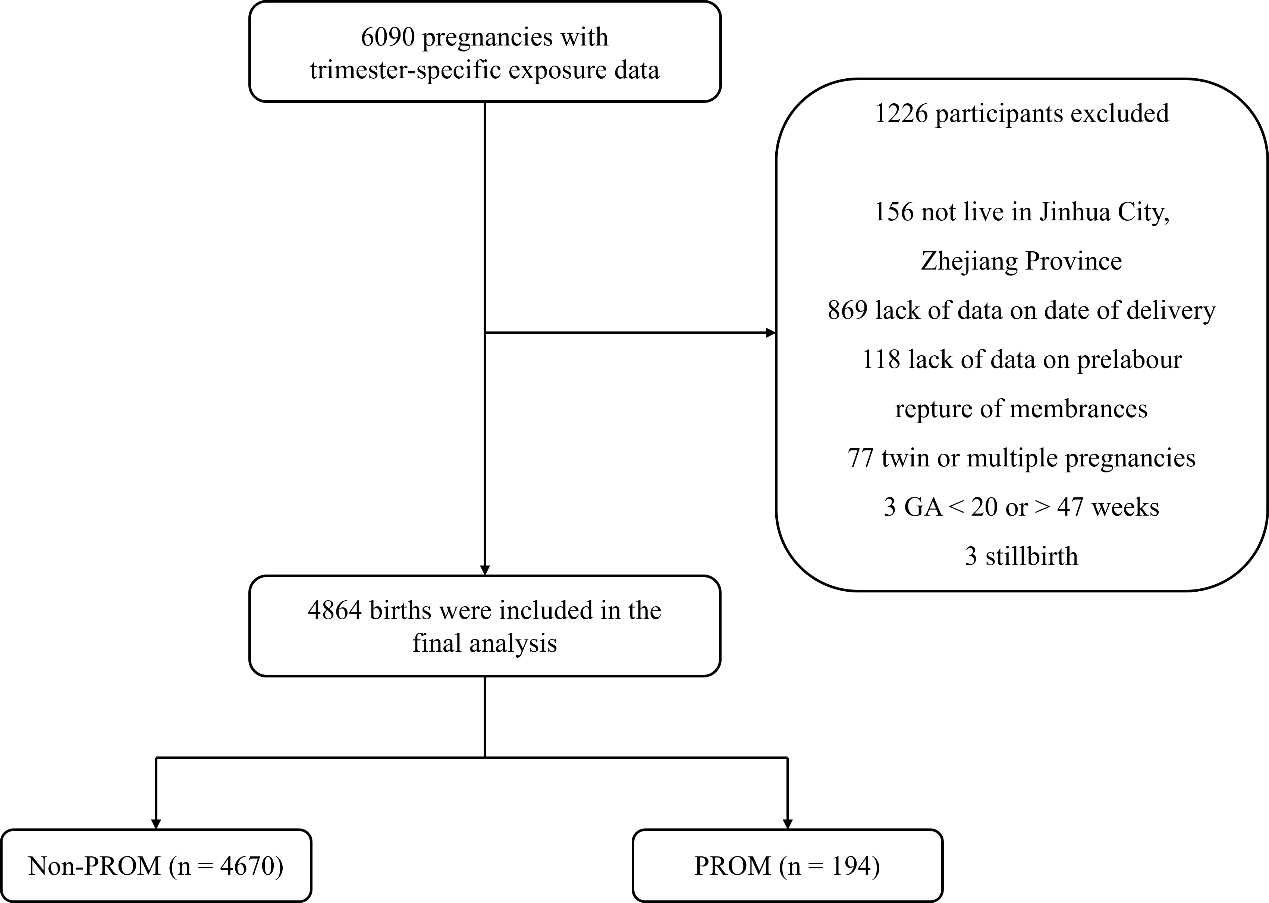


**Fig. S1.** Flowchart of the participants selection.


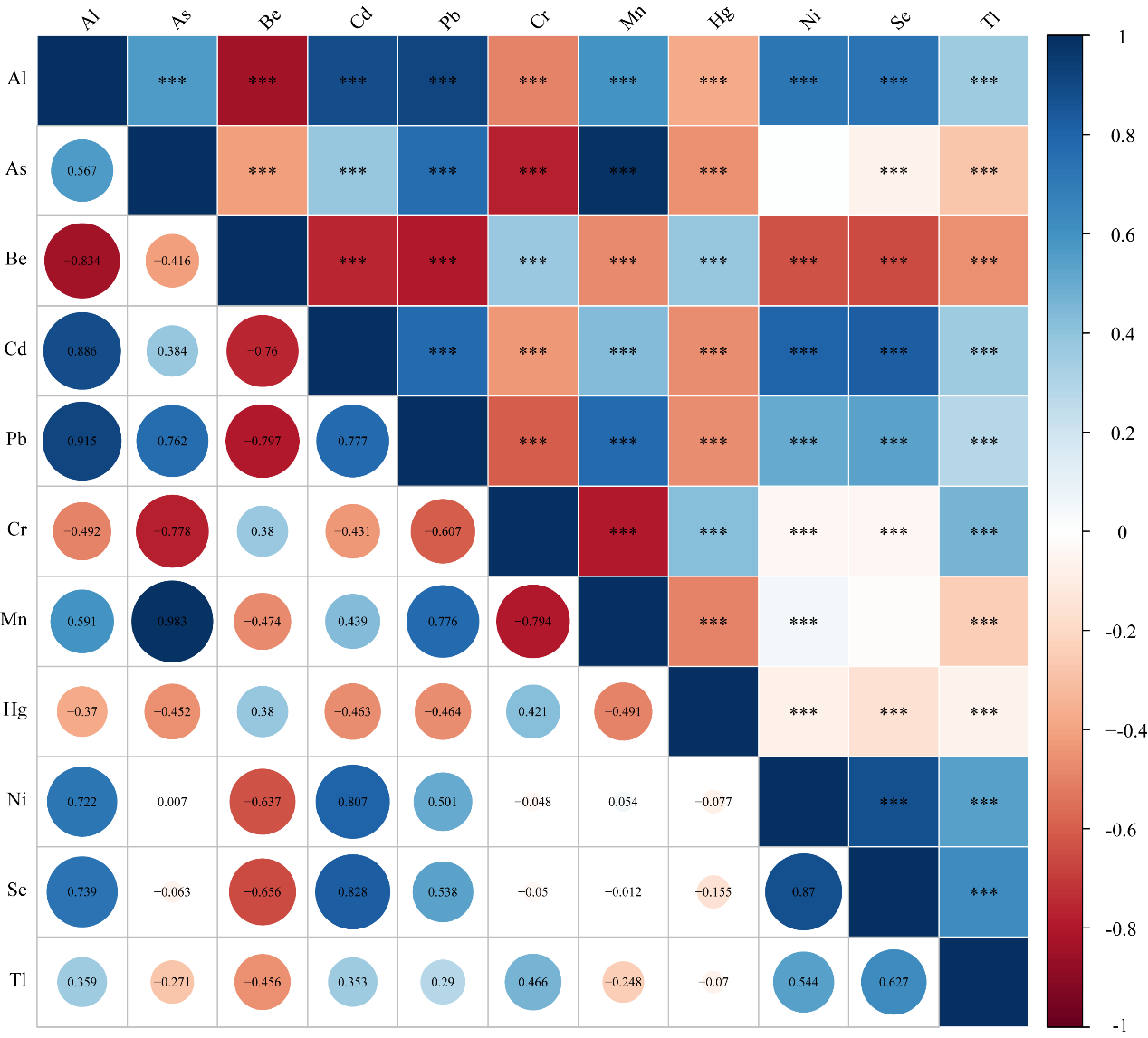


**Fig. S2.** Spearman correlations between metals. Abbreviations: Al, Aluminium; As, Arsenic; Be, Beryllium; Cd, Cadmium; Pb, Plumbum; Mn, Manganese; Cr, Chromium; Hg, Hydrargyrum; Ni, Nickel; Se Selenium; Tl, Thallium.


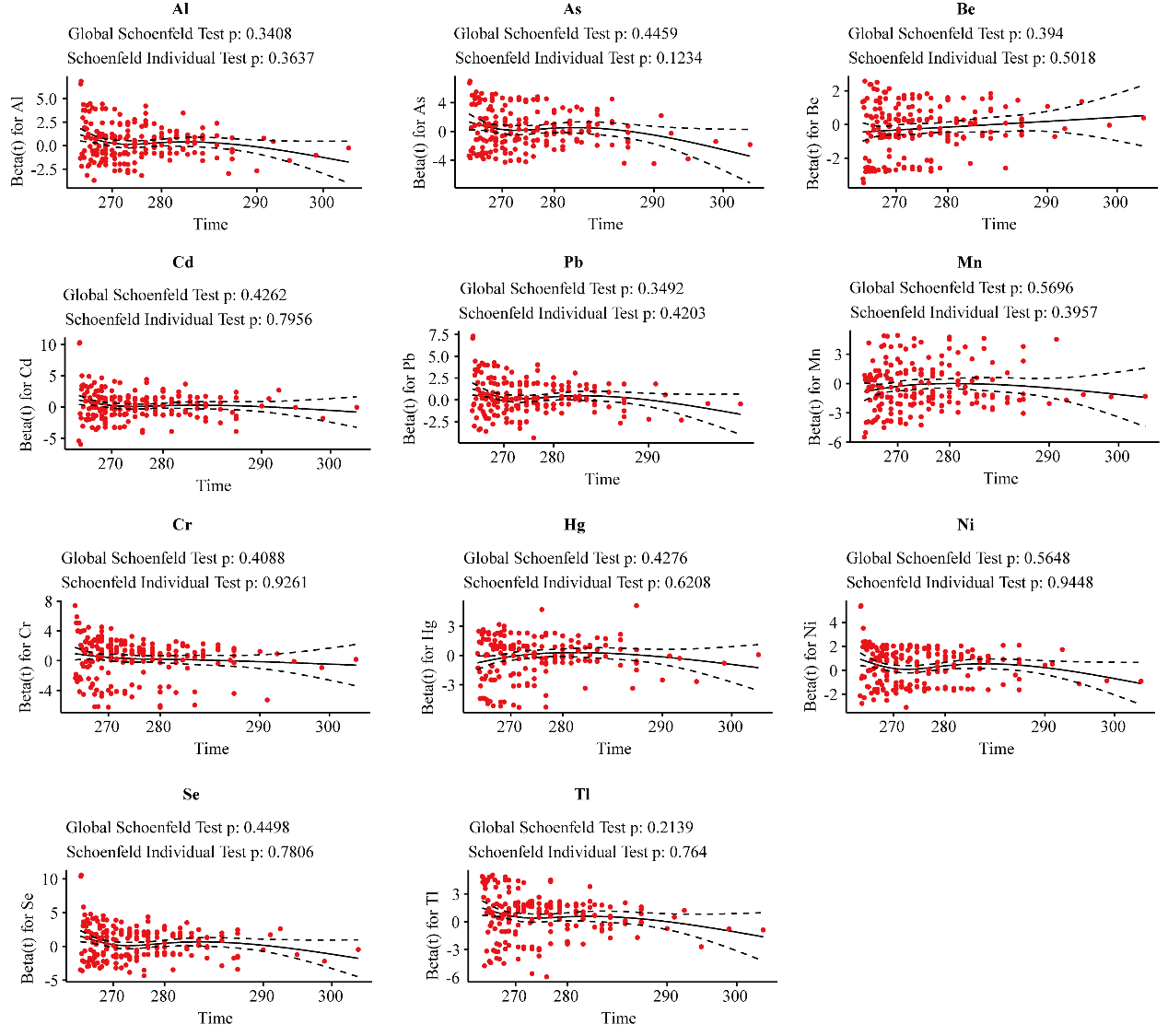


**Fig. S3.** Residual plot for proportional hazards hypothesis test using Schoenfeld residual method. Missing BMI values were not interpolated when performing proportional hazards hypothesis test. Abbreviations: Al, Aluminium; As, Arsenic; Be, Beryllium; Cd, Cadmium; Pb, Plumbum; Mn, Manganese; Cr, Chromium; Hg, Hydrargyrum; Ni, Nickel; Se Selenium; Tl, Thallium.


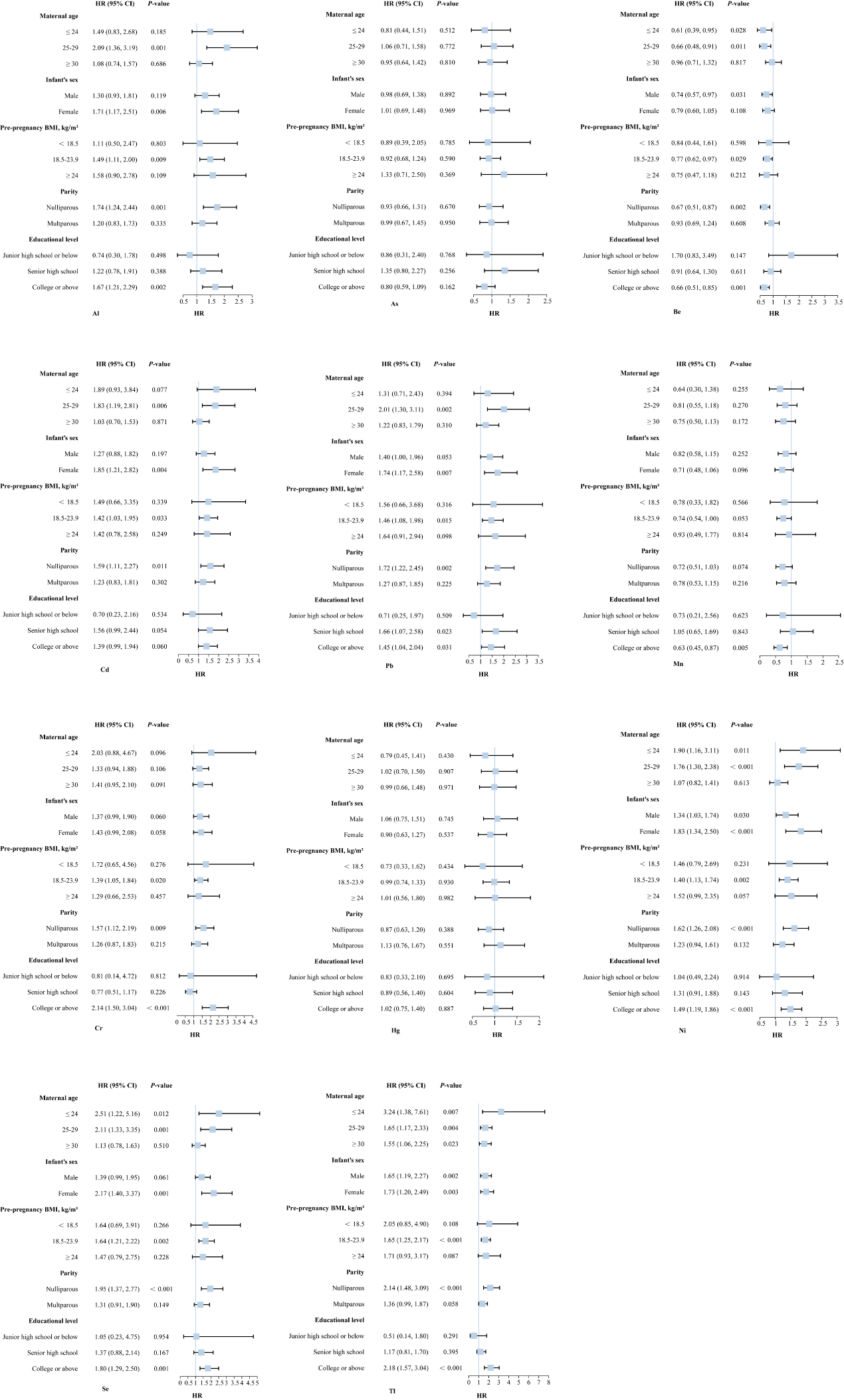


**Fig. S4.** Forest maps for Cox stratified analyses. Effect estimates were hazard ratios (HR) and 95% confidence intervals (95% CI) derived from Cox proportional risk models, with days of pregnancy as the time scale. Standard normal transformations were performed for metal concentrations. The models were adjusted for maternal age, infant’s sex, pre-pregnancy BMI, parity, and maternal educational level. Abbreviations: BMI: body mass index; Al, Aluminium; As, Arsenic; Be, Beryllium; Cd, Cadmium; Pb, Plumbum; Mn, Manganese; Cr, Chromium; Hg, Hydrargyrum; Ni, Nickel; Se Selenium; Tl, Thallium.

**
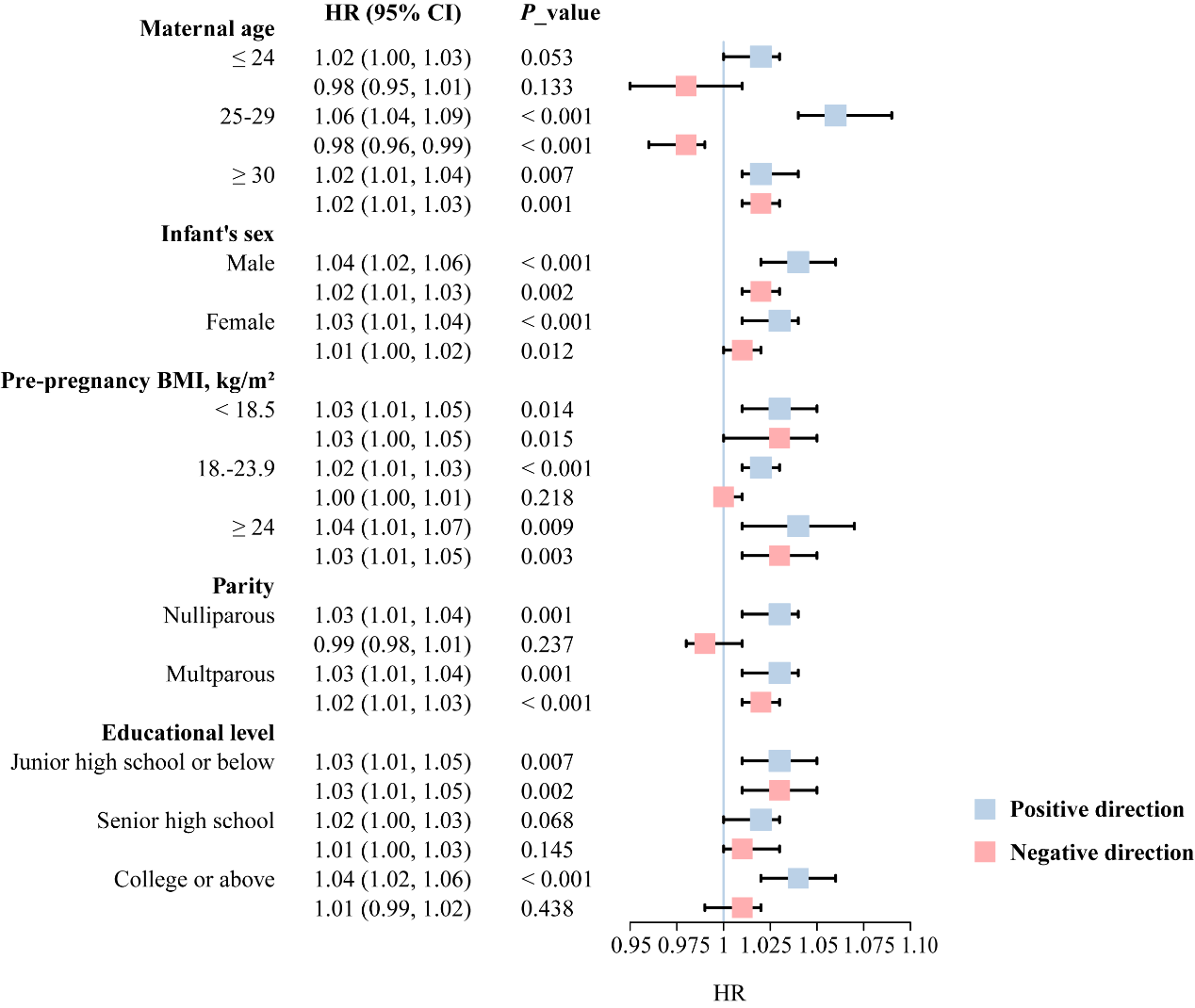
**

**Fig. S5.** Forest maps for GWQS stratified analyses. Effect estimates were odds ratio (OR) and 95% confidence intervals (95% CI) derived from GWQS models. Standard normal transformations were performed for metal concentrations. The models were adjusted for maternal age, infant’s sex, pre-pregnancy BMI, parity, and maternal educational level. Abbreviations: BMI: body mass index; Al, Aluminium; As, Arsenic; Be, Beryllium; Cd, Cadmium; Pb, Plumbum; Mn, Manganese; Cr, Chromium; Hg, Hydrargyrum; Ni, Nickel; Se Selenium; Tl, Thallium.

**References**

[1] Zhang Y, Dong T, Hu W, et al. Association between exposure to a mixture of phenols, pesticides, and phthalates and obesity: Comparison of three statistical models [J]. Environ Int, 2019, 123: 325-36.

[2] Keil A P, Buckley J P, O'Brien K M, et al. A Quantile-Based g-Computation Approach to Addressing the Effects of Exposure Mixtures [J]. Environ Health Perspect, 2020, 128(4): 47004.

[3] Bobb J F, Valeri L, Claus Henn B, et al. Bayesian kernel machine regression for estimating the health effects of multi-pollutant mixtures [J]. Biostatistics, 2015, 16(3): 493-508.

[4] Ye X, Xu T, Yang L, et al. Association between plasma metal exposure and health span in very elderly adults: a prospective cohort study with mixture statistical approach [J]. BMC Geriatr, 2024, 24(1): 388.
